# Effectiveness, acceptability, and potential of lay student vaccinators to improve vaccine delivery

**DOI:** 10.17269/s41997-024-00909-2

**Published:** 2024-07-17

**Authors:** Ryan Yee, Cécile Raymond, Meredith Strong, Lori Seeton, Akash Kothari, Victor Lo, Emma-Cole McCubbin, Alexandra Kubica, Anna Subic, Anna Taddio, Mohammed Mall, Sheikh Noor Ul Amin, Monique Martin, Aaron M. Orkin

**Affiliations:** 1 University of Toronto Emergency First Responders, University of Toronto, Toronto, ON Canada; 2 University Health Network, Toronto, ON Canada; 3 Office of the Vice-Provost, Students, University of Toronto, Toronto, ON Canada; 4 Dalla Lana School of Public Health, University of Toronto, Toronto, ON Canada; 5 Leslie Dan Faculty of Pharmacy, University of Toronto, Toronto, ON Canada; 6 West Toronto Ontario Health Team, Toronto, ON Canada; 7 Department of Family and Community Medicine, University of Toronto, Toronto, ON Canada; 8 Department of Emergency Medicine, St. Joseph’s Health Centre, Unity Health Toronto, Toronto, ON Canada; 9 Li Ka Shing Knowledge Institute of Unity Health Toronto, Toronto, ON Canada

**Keywords:** Vaccines, Task shifting and sharing, Lay health workers, Student-run clinic, Seasonal immunization, Community health planning, COVID-19, Influenza, Vaccins, transfert et partage de tâches, agents de santé profanes, clinique gérée par des étudiants, immunisation saisonnière, planification de la santé communautaire, COVID-19, grippe

## Abstract

**Setting:**

Task sharing can fill health workforce gaps, improve access to care, and enhance health equity by redistributing health services to providers with less training. We report learnings from a demonstration project designed to assess whether lay student vaccinators can support community immunizations.

**Intervention:**

Between July 2022 and February 2023, 27 undergraduate and graduate students were recruited from the University of Toronto Emergency First Responders organization and operated 11 immunization clinics under professional supervision. Medical directives, supported with online and in-person training, enabled lay providers to administer and document vaccinations when supervised by nurses, physicians, or pharmacists. Participants were invited to complete a voluntary online survey to comment on their experience.

**Outcomes:**

Lay providers administered 293 influenza and COVID-19 vaccines without adverse events. A total of 141 participants (122 patients, 17 lay vaccinators, 1 nurse, and 1 physician) responded to our survey. More than 80% of patients strongly agreed to feeling safe and comfortable with lay providers administering vaccines under supervision, had no concerns with lay vaccinators, and would attend another lay vaccinator clinic. Content and thematic analysis of open-text responses revealed predominantly positive experiences, with themes about excellent vaccinators, organized and efficient clinics, and the importance of training, communication, and access to regulated professionals. The responding providers expressed comfort working in collaborative immunization teams.

**Implications:**

Lay student providers can deliver vaccines safely under a medical directive while potentially improving patient experiences. Rather than redeploying scarce professionals, task sharing strategies could position trained lay vaccinators to support immunizations, improve access, and foster community engagement.

**Supplementary Information:**

The online version contains supplementary material available at 10.17269/s41997-024-00909-2.

## Résumé

**Lieu:**

Le partage de tâches peut combler les pénuries de personnels de santé et améliorer l’accès aux soins et l’équité en santé en redistribuant les services de santé vers des prestataires ayant moins de formation. Nous rendons compte des enseignements d’un projet de démonstration visant à déterminer si des vaccinateurs étudiants profanes pourraient appuyer l’immunisation communautaire.

**Intervention:**

Entre juillet 2022 et février 2023, 27 étudiantes et étudiants de premier cycle et de cycles supérieurs ont été recrutés auprès de l’organisation des secouristes opérationnels de l’Université de Toronto pour gérer 11 cliniques de vaccination sous la supervision de personnel spécialisé. Des directives médicales, appuyées par une formation en ligne et en présentiel, ont permis à ces prestataires profanes d’administrer des vaccins et de les consigner en dossier sous la supervision d’infirmières, de médecins ou de pharmaciens. Les personnes participantes ont été invitées à répondre à un sondage en ligne sur leur expérience.

**Résultats:**

Les prestataires profanes ont administré 293 vaccins contre la grippe et la COVID-19 sans manifestations postvaccinales indésirables. En tout, 141 personnes (122 patients, 17 vaccinateurs profanes, 1 infirmière et 1 médecin) ont répondu au sondage. Plus de 80 % des patients ont dit se sentir tout à fait en sécurité et à l’aise de recevoir des vaccins administrés par des prestataires profanes sous supervision, n’avoir aucune inquiétude vis-à-vis des vaccinateurs profanes et être disposés à se présenter à une autre clinique gérée par des vaccinateurs profanes. L’analyse du contenu et des thèmes des réponses aux questions ouvertes a révélé des expériences majoritairement positives, et des thèmes axés sur l’excellence des vaccinateurs, l’organisation et l’efficacité des cliniques, ainsi que l’importance de la formation, des communications et de l’accès à des professionnels réglementés. Les prestataires ayant répondu au sondage se sont dit à l’aise de travailler au sein d’équipes de vaccination collaboratives.

**Conséquences:**

Des prestataires étudiants profanes peuvent administrer des vaccins en toute sécurité en suivant une directive médicale, et cela peut potentiellement améliorer l’expérience des patients. Plutôt que de redéployer des ressources professionnelles limitées, les stratégies de partage de tâches pourraient placer des vaccinateurs profanes formés pour appuyer l’immunisation, améliorer l’accès et favoriser l’engagement communautaire.

## Introduction

Enhancing vaccine access, delivery, uptake, and equity remains a significant challenge (Lazarus et al., 2022). In Canada, vaccines are routinely administered by regulated health providers such as nurses, physicians, and pharmacists. While this model ensures professional oversight, this can also create healthcare strain by imposing substantial demand on health human resources, particularly during seasonal and pandemic immunization campaigns (Kholina et al., 2022; Sell et al., 2021).

Task sharing offers potential strategies to alleviate healthcare strain while enhancing vaccine access, delivery, and uptake (Gibson et al., 2023; Lewin et al., 2005). Task sharing approaches involve the deliberate redistribution of healthcare services to individuals with less extensive training, such as peers, paraprofessionals, or lay health workers (Gibson et al., 2023; Lewin et al., 2005; Orkin et al., 2019). Training and authorizing lay health workers to administer vaccines can expand the vaccinator workforce, bring services closer to communities, and optimize the skills of scarce professionals, including nurses, physicians, and pharmacists, who can then assume supervisory or leadership roles and provide more specialized services (Gibson et al., 2023; Orkin et al., 2021). In December of 2021, the Ontario government made an amendment to the *Regulated Health Professions Act* which enabled any person to administer COVID-19 vaccines when authorized and supervised by a regulated nurse, physician, or pharmacist (*Regulated Health Professions *Act, 1991). Intramuscular injection is a basic procedure with minimal training and dexterity requirements that provides a unique opportunity for task sharing. Many jurisdictions already use written medical directives to delegate intramuscular injection (CPSO Delegation of Controlled Acts, 2023) (Supplementary Resource 1). For example, medical directives can enable paramedics and first-aiders to administer intramuscular epinephrine for anaphylaxis and naloxone for opioid overdose—medical conditions and environments with greater complexity and acuity than a typical vaccine clinic. In reality, many individuals have the willingness, responsibility, and technical skills required to become a vaccinator, but may lack access to formal training, supervision, or infrastructure required to assist in practice (Bourgeault et al., 2020). Rather than constraining vaccine administration to the scarce Canadian healthcare workforce facing shortages and burnout (Canadian Medical Association, 2021), principles of task sharing may empower a broader cadre of lay or paraprofessional providers to administer vaccines with appropriate motivation, training, supervision, and authorization. Such a paradigm shift could revolutionize the vaccination landscape by truly engaging communities in their own care.

The use of lay providers in vaccine administration is not new. Globally, lay health workers have administered immunizations for decades (Gibson et al., 2023; Lewin et al., 2005). Interventions using lay health workers have been shown to increase immunization uptake, reduce morbidity, and improve patient outcomes in several populations (Juon et al., 2016; Krieger et al., 2000; Lewin et al., 2005). While many lay health worker immunization campaigns involve patient education and outreach, there are few published studies that examine the utility and acceptability of positioning lay providers as vaccinators (Gibson et al., 2023).

At least 20 different countries have engaged lay health workers as vaccinators using national programs or research studies since the year 2000, predominantly in low- and middle-income countries (Gibson et al., 2023; Orkin et al., 2019). In June of 2021, New Zealand introduced the COVID-19 Vaccinator Working Under Supervision (CVWUS) program to authorize non-regulated health providers to administer vaccines, illustrating task sharing on a nationwide scale (Unregulated Healthcare Professionals, 2023). Research on the feasibility, potential, and acceptance of a lay vaccinator model in Canada may lead to more efficient care and optimized use of health human resources.

To assess the effectiveness of a task sharing model involving lay providers in urban Toronto, we formed a multi-institutional collaboration with university students and faculty, healthcare providers, hospital staff and leaders, and community partners to provide a series of COVID-19 and influenza vaccination clinics with lay university students as vaccinators (lay vaccinators). An online research survey was administered at clinics to evaluate perspectives from patients and providers on the use of lay vaccinators.

## Intervention

### Setting

Between July 2022 and February 2023, the University of Toronto Emergency First Responders (UTEFR) collaborated with the Dalla Lana School of Public Health, the University Health Network (UHN), and the West Toronto Ontario Health Team to initiate 11 COVID-19 and influenza immunization clinics involving lay vaccinators. Clinics operated across three settings in Toronto, including (1) a downtown university-commons building open to students and the public; (2) a university residence building primarily occupied by undergraduate students; and (3) three different off-campus community health centres in West Toronto. The clinics on campus operated with no more than ten lay vaccinators per 4–8-h clinic; at least one physician and one nurse to supervise the clinic and train vaccinators; and one to two hospital-affiliated administrative staff to support registration. At the community health clinics off-campus, both UTEFR lay vaccinators and nursing students from Toronto Metropolitan University administered vaccines under the supervision of two physicians. Lay vaccinators served as volunteers for this project and received a one-time honorarium ($40 CAD) to provide reimbursement for food/travel costs. The University of Toronto Students’ Union provided students with access to general liability insurance coverage for their volunteer role. All other staff were remunerated by their respective organizations and supervisors required their own professional liability insurance.

### Vaccinator selection and training

UTEFR is a volunteer student organization that provides pre-hospital care and first-aid education on and around the downtown University of Toronto St. George campus. Membership is open year-round to undergraduate and graduate students with healthcare backgrounds ranging from no experience, to varying first-aid/responder certifications, to formal health professional training. All students at the University of Toronto were therefore eligible to join UTEFR, complete training, and become vaccinators for this project. UTEFR comprised more than 80 members at the time of clinic offerings, though only approximately 30 were “active members” and consistently participated in UTEFR trainings and events. Vaccinator training was offered to all members, of whom 30 expressed interest and created a COVID Care eLearning account for asynchronous training, and 27 students completed all training requirements and served as vaccinators (90% completion).

Students were required to complete four steps of vaccinator training at their own pace:

1. “Vaccinator course” — COVID Care Learning (online asynchronous)
2. “Vaccine Clinic Admin course” — COVID Care Learning (online asynchronous)
3. Pre-clinic training with UTEFR Executive Director (online synchronous)
4. Hands-on training and authorization with clinic lead and onsite supervisors (in-person at clinics)

The blended online/in-person training model provided vaccinators with both structure and flexibility in meeting learning requirements during university studies. The asynchronous COVID Care Learning courses were designed by interprofessional experts at the UHN-affiliated Michener Institute to support the role optimization and rapid redeployment of various health providers (e.g., nursing/medicine/pharmacy/physiotherapy/nutrition/social work) during the pandemic (COVID Care Learning About Us, 2024). The “COVID Vaccinator” and “COVID Admin” modules provided videos and resources on how to perform intramuscular injections (~ 2.5 h) and document in the Ontario COVID immunization record (~ 2 h) (COVID & Critical Care Learning, 2020). These modules additionally contained information on clinic roles/responsibilities, consent/eligibility, and cybersecurity. Online synchronous training was conducted as a 1-h virtual session with the UTEFR Executive Director, reviewing the medical directive and clinic logistics, and discussing communication strategies and professionalism (Professional Practice Behaviour for All Health Professional Students, 2015). Our dynamic interprofessional training model evolved to further incorporate a pharmacist-led seminar on the Comfort, Ask, Relax, Distract (CARD) approach to managing vaccine-associated pain (Taddio et al., 2022) as well as collaborative training and clinic operation with nursing students from the Toronto Metropolitan University.

In-person training took place at clinics with the supervising professionals (physician/nurse/pharmacist) and designated clinic lead (lay vaccinator with immunization/first-aid experience), which incorporated both a workshop-model with fake skin/oranges and scenario-based learning. Students were required to demonstrate competence and confidence in deltoid landmarking, administration procedure, documentation, and following all procedures in the medical directive to become vaccinators (Supplementary Resource 1). This training and authorization allowed UTEFR members to administer COVID-19 and influenza vaccines only at UTEFR-affiliated vaccine clinics and did not confer authorization in any other setting or context. In addition to the medical directives, lay vaccinators were required to adhere to the University of Toronto Code of Student Conduct (Code of Student Conduct, 2019) and follow the Standards of Professional Practice Behaviour for all Health Professional Students (Professional Practice Behaviour for All Health Professional Students, 2015) when representing UTEFR both on and off campus.

### Supervision

Lay vaccinators engaged as members of an interdisciplinary healthcare team with multiple levels of supervision. In alignment with policies set by the Regulated Health Professions Act, our clinics required that supervisors had licensure to practice in Ontario as a nurse (RN/RPN/NP), physician (MD), or pharmacist (PharmD). All UTEFR clinics were supervised by at least one physician and one nurse to prepare vaccines, address questions from patients and lay vaccinators, and respond to potential emergencies. Regulated providers were also responsible for vaccinating individuals under the age of 16, complex or challenging populations, or anyone else who requested a regulated provider. UTEFR members worked in vaccinator-documenter dyads to enhance accountability, learning, and peer supervision. Vaccine stations were also positioned within view and earshot of supervisors where vaccines were being prepared, and private spaces were available upon request. The clinic lead with previous first-aid/vaccinator experience was designated at each clinic to support training, provide additional clinic monitoring and peer supervision, rapidly relay information to regulated supervisors, and provide administrative support for regulated supervisors as needed.

### Evaluation

A voluntary research survey (Supplementary Resource 2) was created and administered using the Qualtrics survey platform (Qualtrics Provo UT) to evaluate perceptions and experiences regarding lay vaccinators. Questions were designed to collect both quantifiable and qualitative assessments of participant comfort levels, safety perceptions, and overall satisfaction with the lay vaccinator-operated clinic. Eligible clinic attendees were invited to complete the survey in the post-vaccination waiting area; a separate section of the survey was available to lay vaccinators and regulated professionals with additional questions about their experience administering vaccines or supervising vaccinators. Voluntary surveys were distributed at seven of the 11 clinics; surveys were not available for administration at the initial two clinics, and two later clinics occurred off campus and could not distribute surveys due to staffing limitations.

Likert scales (5-point agreement) and binary (yes/no) questions were primarily used to assess comfort levels, safety perceptions, and acceptance of the clinical model, while open-text questions aimed to gather qualitative feedback on experiences, concerns, and suggestions for improvement. Likert and binary response data were reported as descriptive statistics, while content and thematic analysis was used to interpret and summarize qualitative responses from open-text questions. For this analysis, two authors independently assessed the content of each response and assigned a code of positive, neutral, constructive, or negative. Inductive descriptive coding was used to develop thematic codes, which had a high degree of consensus in the initial categorization and no unresolvable conflicts when developing themes. This project was conducted in accordance with the ethical standards of the University of Toronto Research Ethics Board (Human Ethics Protocol #43500).

## Outcomes

Lay student vaccinators administered a total of 293 influenza and COVID-19 vaccines across 11 UTEFR immunization clinics. In total, 203 COVID-19 and 90 influenza vaccines were administered to 253 participants, 40 of whom received both a flu and a COVID-19 vaccine at the same time. A total of 91 vaccines were administered at four clinics where the survey was not initiated (two on-campus and two off-campus clinics). In total, 162 patients were eligible to complete the survey. A total of 157 responses were received, of which 16 were excluded for incompleteness (90% completion). Of the 141 complete submissions, 122 were from clinic patients (75% response), 17 from lay vaccinators (63% response), and two from supervising professionals (20% response) (Supplementary Resources 3 and 4). No medication errors or adverse events occurred.

**Fig. 1:**
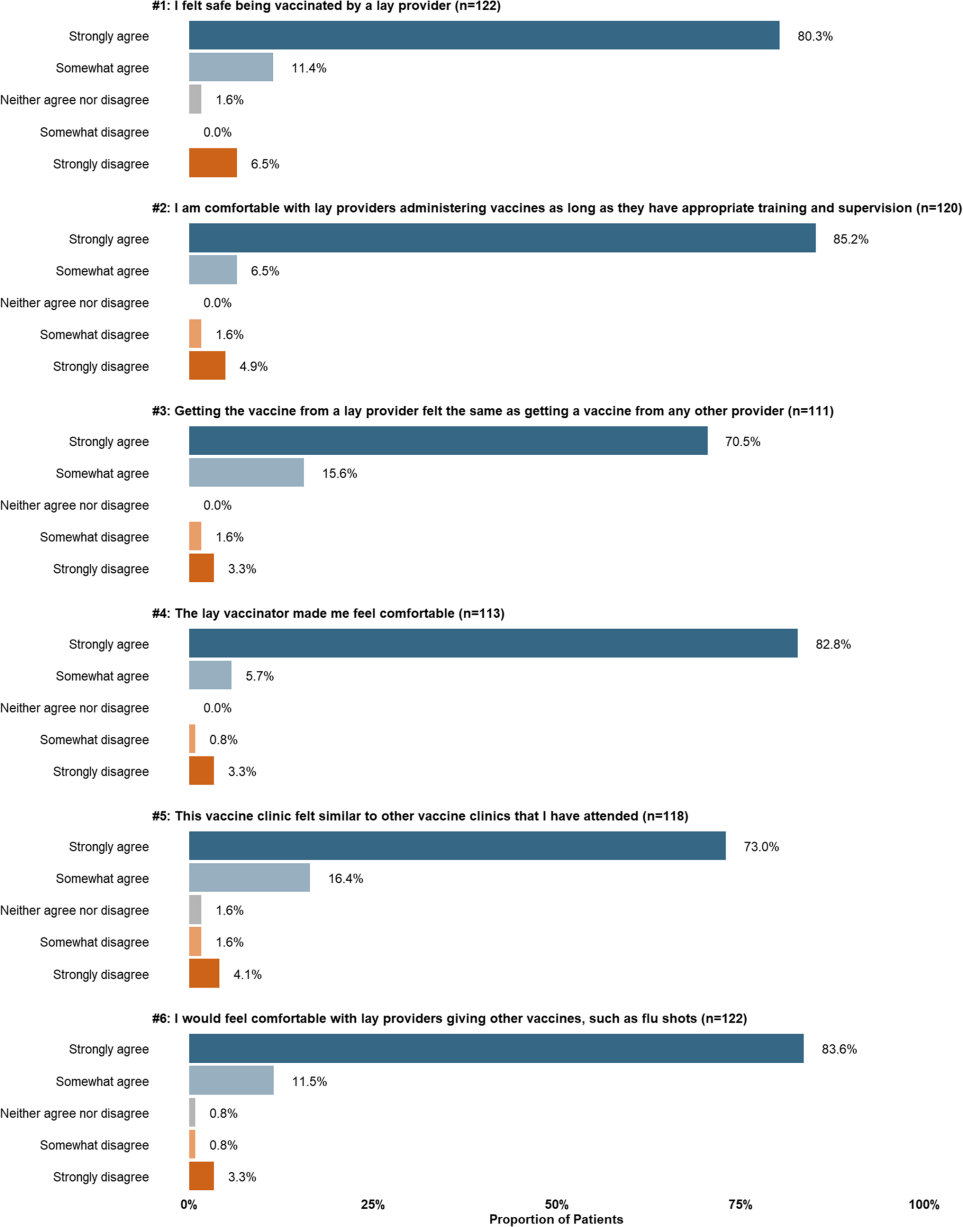
Proportion of patients’ reported level of agreement per statement (*n* = 122). Patients were not required to provide a response to every statement

Patient Likert scale responses were very supportive, with at least 70% of patients indicating “strongly agree” to all statements (5-point agreement positively worded) which examined perceptions of safety, comfort, and similarity to other clinics (Fig. 1).

**Fig. 2:**
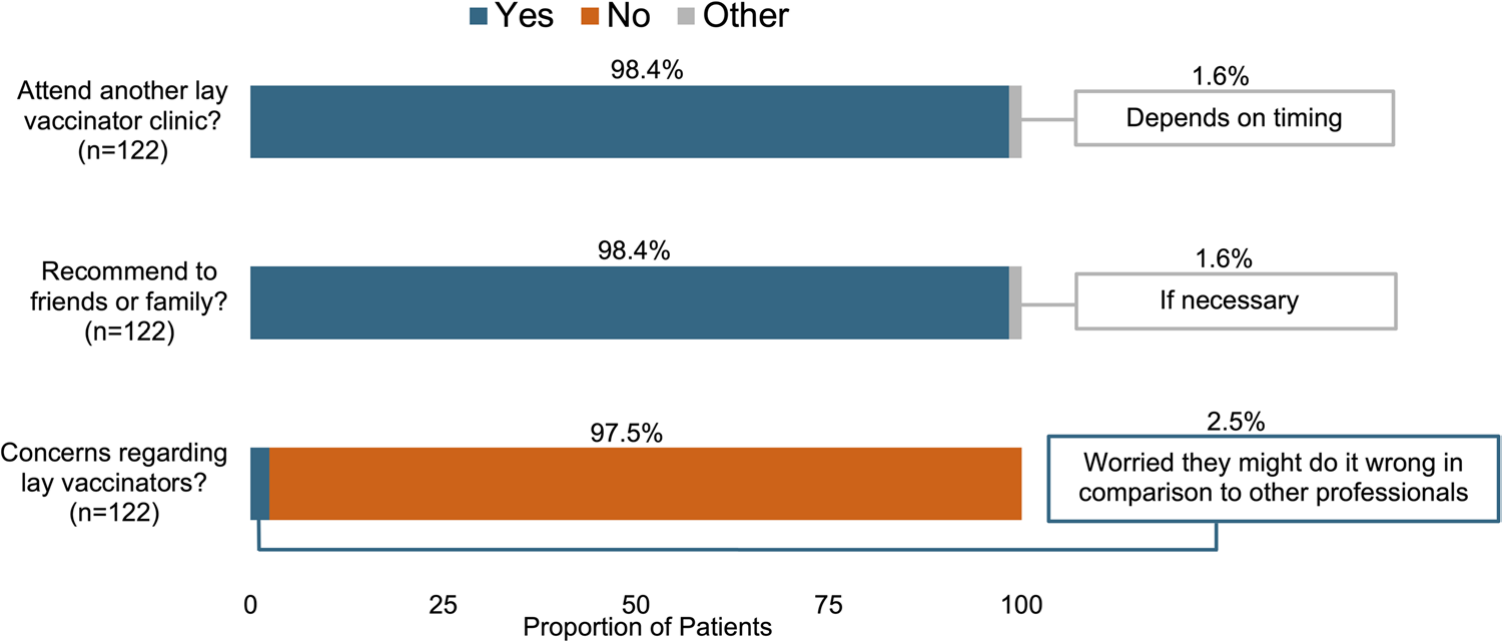
Patient responses to yes/no/other questions with quotes from open-text responses

All 122 patients answered the binary yes/no questions, with 98% indicating they would attend another clinic operated by lay vaccinators, 98% willing to recommend this clinic to friends or family, and 98% having no concerns with lay vaccinators (Fig. 2). Of the 3% (*n* = 3 patients) who indicated yes to having concerns, one expressed worry that lay vaccinators may incorrectly administer the vaccines compared to professionals (Fig. 2).

**Fig. 3:**
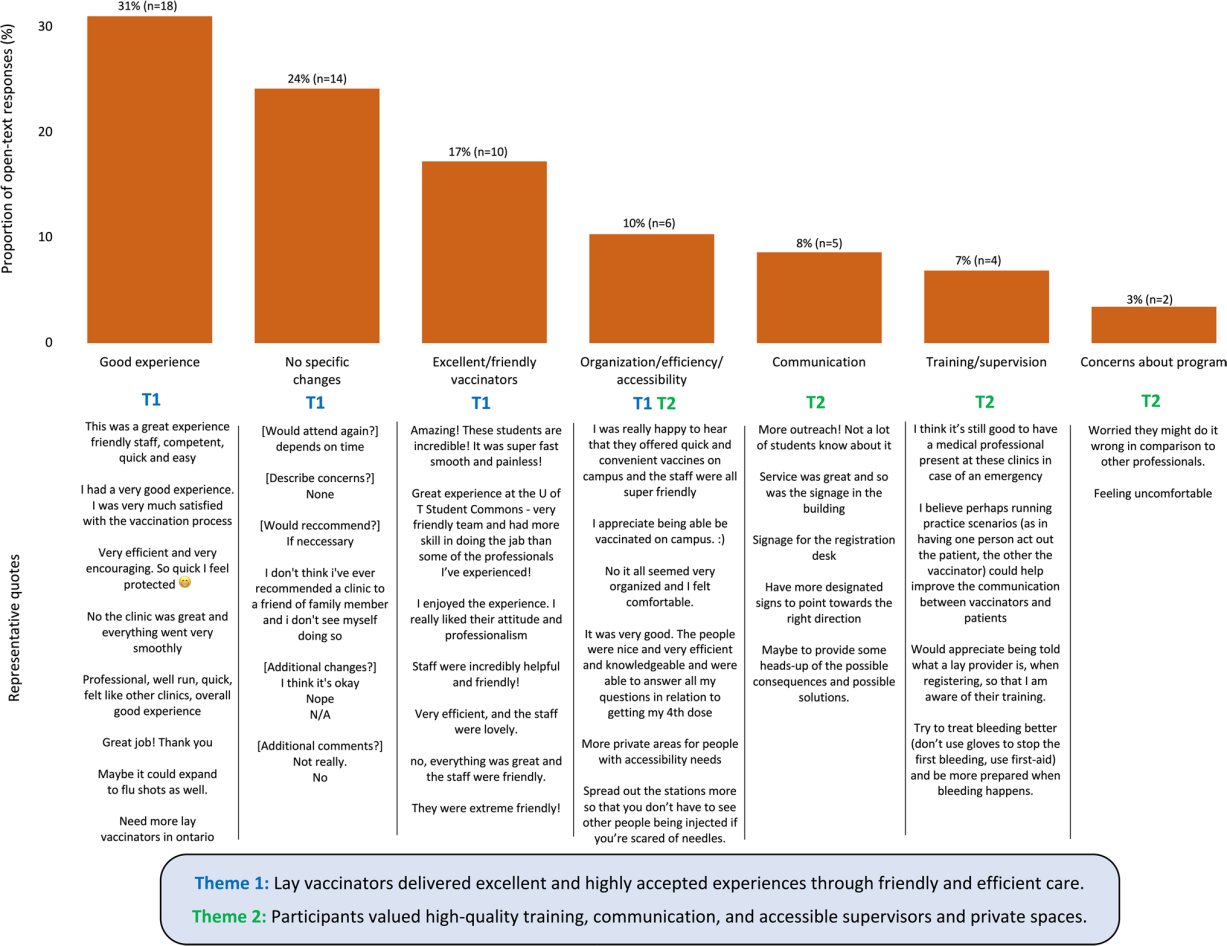
Thematic analysis of open-text responses to lay vaccinator clinic survey (*n* = 59)

The survey received 59 responses to its four open-text questions, which asked participants to describe their experience at the clinic, whether they had any concerns with the clinic, whether they would make any changes to the clinic, and whether they had any additional comments they would like to provide. Content analysis revealed predominantly positive (*n* = 30) and neutral (*n* = 17) responses, with some constructive responses with feedback for future clinics (*n* = 10), and minimal negative responses expressing dissatisfaction (*n* = 2) (Table 1). Inductive descriptive coding from two independent authors resulted in seven new codes that could classify open-text quotes for thematic analysis. Consensus from iterative discussions led to the creation of two overarching themes, which indicated that lay vaccinators delivered highly effective and acceptable services through friendly and efficient care, and that participants valued high-quality training, communication, and access to supervision and private spaces (Fig. 3).

**Table 1:** Content analysis of open-text responses to lay vaccinator clinic survey (*n* = 59)

Feedback category	Summary of qualitative responses	Quotes
Positive (*n* = 30)	Respondents felt safe and protected; that the clinic improved their vaccination experience; vaccinators were professional and friendly; the clinic was accessible, efficient, and smooth; and more lay vaccinators were needed	“This was a great experience friendly staff, competent, quick and easy” "Very efficient and very encouraging. So quick I feel protected" “Amazing! These students are incredible! It was super fast smooth and painless!” “The vaccine clinic is good”
Neutral (*n* = 17)	Individuals thought the experience was comparable to previously attended clinics; had no concerns with the service; had neither a good nor bad experience; and would attend again or recommend the clinic if needed	“I think it’s okay” “If necessary” [would recommend?] “Not really” [additional feedback?]
Constructive (*n* = 10)	Participants wanted more outreach and signage; active measures to reduce potential physical/emotional discomfort; access to regulated providers and private space when needed; high-quality training and communication of qualifications and vaccine information	"I think it’s still good to have a medical professional present at these clinics in case of an emergency (e.g. bad reaction to the vaccine or anything else that might not have fallen under the lay provider’s training)" “More private areas for people with accessibility needs" “I think I was in the system so no one checked my health card, but overall it was pretty quick and maybe more discussion on what dose I got last time and information on the two types of bivalent would have been helpful.”
Negative (*n* = 2)	Participants reported feelings of discomfort or worried that lay providers may do it wrong compared to regulated professionals	“Feeling uncomfortable” “Worried they might do it wrong in comparison to other professionals.”

Lay providers also had an opportunity to fill out a voluntary survey rating their comfort administering the vaccine, the training, and supervision. Of the 27 lay vaccinators who participated in clinics, 17 completed the survey. Of the lay vaccinator respondents, 76% had no previous experience as a vaccinator. When rating comfort levels on a Likert scale, 94% of lay vaccinators either strongly or somewhat agreed that they felt comfortable administering the vaccine. Additionally, 94% strongly or somewhat agreed that the amount of training they received was enough to instil confidence in administering the vaccines. Respondents worked well in an interdisciplinary team, as 88% of lay vaccinators strongly or somewhat agreed that the supervising staff were approachable and able to answer questions. Finally, 100% of responding lay vaccinators agreed that they would feel comfortable getting vaccinated by another lay provider.

Two supervising professionals completed the survey and rated their feelings of comfort and safety in supervising a cadre of lay vaccinators on a Likert scale. Both responding supervisors strongly agreed they felt comfortable working with and supervising lay vaccinators; the training provided to lay vaccinators was sufficient; they would feel comfortable supervising more lay vaccinators at a future clinic; and lay providers should be able to work at any vaccine clinic provided they have appropriate training. One health professional noted that they would feel comfortable supervising up to ten lay vaccinators; the other health professional stated that provided lay vaccinators had the appropriate training, they would be comfortable supervising any number of lay vaccinators in their practice.

## Discussion

Task sharing strategies involving lay student vaccinators can be safe and effective, and can create positive patient experiences. Lay vaccinators rapidly deployed and operated their own COVID-19 and influenza vaccine clinics under supervision and integrated with existing healthcare teams including nursing students to support immunization delivery. The Likert scales and binary responses revealed a strong majority of patients who felt safe and comfortable with lay vaccinators. Open-text responses revealed predominantly positive experiences about friendly and professional vaccinators, organized and efficient clinics, and the importance of communication, training, and access to supervision and private spaces.

Although our project engaged lay vaccinators as volunteers, our goal was not to demonstrate the viability of volunteer vaccinators as a potential strategy to address health human resource challenges. Rather, this project was solely about demonstrating that lay providers with minimal essential training can deliver vaccines safely, effectively, and uneventfully under medical directive, or through enabling legislation. This is in itself a substantial innovation given how few vaccines were given by lay providers in Canada during the pandemic, even in the face of massive healthcare worker shortages and burnout (Canadian Medical Association, 2021). Since our project was not intended to identify an optimal ratio between health professionals and lay vaccinators, the variance between 10 and any larger number of supervisees is perhaps more reflective of a general willingness and comfort supervising a relatively large number of lay vaccinators rather than an actual difference in the acceptable upper limit of supervised lay vaccinators. At a systems level, lay vaccinators could be engaged through a variety of models, including paid, voluntary, or even paraprofessional development. One lay vaccinator option could be to engage volunteers, but the complexity of sustainable volunteer-based systems has been well documented and additional considerations would be necessary to address retention and role satisfaction (Tetui et al., 2023). Other options could include the development of a novel paid lay vaccinator or paraprofessional role trained and employed specifically for this purpose—a potentially valuable, rewarding, and sustainable new member of the health workforce team.

While this project found early success in the university student context, it was not designed to compare lay vaccinators directly with regulated health providers. Many patients fell into the same age category as vaccinators and it is less clear whether lay vaccinators would be as effective beyond a perceivable “peer-vaccinator” context. Moreover, the survey was not designed to evaluate baseline participant fears or reluctance towards vaccination, which could have also shaped perceptions about lay vaccinators. Nevertheless, our pilot findings agreed with earlier studies on the acceptability of pharmacists delivering vaccines during a time where pharmacists did not regularly conduct immunizations (MacDougall et al., 2016). While lay vaccinators may be able to support seasonal and pandemic immunization campaigns, it should be noted that routine immunization schedules during checkups with primary care providers can offer benefits beyond vaccination, such as disease screening and counselling. Although there were no adverse events across 293 doses, a larger sample size and longer-term surveillance may be necessary to detect rare phenomena. Longer-term follow-up studies with lay providers could investigate whether becoming a vaccinator had transformative impacts or influenced vaccinators’ professional development. Though it was not our primary intent to assess financial outcomes, the honorarium provided to volunteers was substantially lower than standard Ontario professional rates, highlighting potential for improved cost-effectiveness and pay equity; additional compensation may therefore be appropriate for professionals who have increased supervisory responsibilities and administrative duties (Office of the Auditor General of Ontario, 2022).

## Conclusion

Intramuscular vaccines can be safely administered by lay vaccinators with high levels of acceptance by patients and providers. The success of this model relied on the collective agreement of patients, lay vaccinators, and regulated providers including nurses, physicians, and pharmacists, evidenced by overwhelmingly supportive feedback. Collaborative healthcare teams can leverage directives and delegation to optimize roles and share responsibilities to potentially improve patient care while reducing burden. The small proportion of individuals with apprehensions reflects the ongoing need for onsite supervision and access to regulated providers when needed. While acknowledging the need for further research, this study provides valuable insights into broadening the spectrum of vaccinators in a collaborative, community-centric care landscape that reinforces task sharing strategies and patient involvement as foundations to promoting healthcare accessibility and trust, and improving public health outcomes.

### Implications for policy and practice

What are the innovations in this program?

- We demonstrate the first known immunization clinics in Toronto, ON, to train lay university students as vaccinators.
- Using online/in-person training and medical directives, lay vaccinators could integrate with existing healthcare teams involving physicians, pharmacists, nurses, and nursing students to support community immunizations with high levels of patient safety, comfort, and acceptability.

What are the burning research questions for this innovation?

- A larger sample size and longer-term follow-up studies are necessary to ensure that safety statistics are valid and can be generalized to a greater scale and to the general population.
- Future studies should recruit patients and vaccinators from a diverse selection of settings to study outcomes outside the student/peer-vaccinator context (e.g., training community leaders as vaccinators to enhance access and uptake in marginalized communities or equity-seeking groups).
- A clinical trial could optimally compare lay vaccinators to regulated providers to examine patient safety and acceptance, changes to access, efficiency, and financial outcomes, and develop strategies to help promote pay equity.

## Supplementary Information

Below is the link to the electronic supplementary material.

Supplementary file1 (PDF 139 KB)

Supplementary file2 (PDF 160 KB)

Supplementary file3 (PDF 206 KB)

Supplementary file4 (PDF 129 KB)

## Data Availability

All data supporting the findings of this study are available upon request to the corresponding author. A full list of survey questions is provided in Supplementary Resource 2.

## References

[CR1] Bourgeault, I. L., Maier, C. B., Dieleman, M., Ball, J., MacKenzie, A., Nancarrow, S., Nigenda, G., & Sidat, M. (2020). The COVID-19 pandemic presents an opportunity to develop more sustainable health workforces. *Human Resources for Health,**18*(1), 83. 10.1186/s12960-020-00529-0 33129313 10.1186/s12960-020-00529-0 PMC7602762

[CR2] Canadian Medical Association. (2021). *CMA 2021 National Physician Health Survey*. Retrieved January 2024, from https://www.cma.ca/sites/default/files/2022-08/NPHS_final_report_EN.pdf

[CR3] Code of Student Conduct. (2019). University of Toronto Office of the Governing Council. Retrieved October 8, 2023, from https://governingcouncil.utoronto.ca/secretariat/policies/code-student-conduct-december-13-2019

[CR4] COVID and Critical Care Learning. (2020). The Michener Institute. Retrieved March 7, 2023, from https://michener.ca/criticalcarelearning/

[CR5] COVID Care Learning About Us. (2024). The Michener Institute. Retrieved March 27, 2024, from https://criticalcarelearning.ca/local/staticpage/view.php?page=aboutUs

[CR6] CPSO Delegation of Controlled Acts. (2023). College of Physicians and Surgeons of Ontario. Retrieved September 30, 2023, from https://www.cpso.on.ca/Physicians/Policies-Guidance/Policies/Delegation-of-Controlled-Acts

[CR7] Gibson, E., Zameer, M., Alban, R., & Kouwanou, L. M. (2023). Community health workers as vaccinators: A rapid review of the global landscape, 2000–2021. *Global Health: Science and Practice,**11*(1), e2200307. 10.9745/GHSP-D-22-00307 36853637 10.9745/GHSP-D-22-00307 PMC9972374

[CR8] Juon, H.-S., Strong, C., Kim, F., Park, E., & Lee, S. (2016). Lay health worker intervention improved compliance with hepatitis B vaccination in Asian Americans: Randomized controlled trial. *PLoS ONE,**11*(9), e0162683. 10.1371/journal.pone.0162683 27617742 10.1371/journal.pone.0162683 PMC5019387

[CR9] Kholina, K., Harmon, S. H. E., & Graham, J. E. (2022). An equitable vaccine delivery system: Lessons from the COVID-19 vaccine rollout in Canada. *PLoS ONE,**17*(12), e0279929. 10.1371/journal.pone.0279929 36584230 10.1371/journal.pone.0279929 PMC9803301

[CR10] Krieger, J. W., Castorina, J. S., Walls, M. L., Weaver, M. R., & Ciske, S. (2000). Increasing influenza and pneumococcal immunization rates: A randomized controlled study of a senior center-based intervention. *American Journal of Preventive Medicine,**18*(2), 123–131. 10.1016/s0749-3797(99)00134-8 10698242 10.1016/s0749-3797(99)00134-8

[CR11] Lazarus, J. V., Wyka, K., White, T. M., Picchio, C. A., Rabin, K., Ratzan, S. C., Parsons Leigh, J., Hu, J., & El-Mohandes, A. (2022). Revisiting COVID-19 vaccine hesitancy around the world using data from 23 countries in 2021. *Nature Communications,**13*(1), 1. 10.1038/s41467-022-31441-x 10.1038/s41467-022-31441-x PMC9247969 35778396

[CR12] Lewin, S. A., Dick, J., Pond, P., Zwarenstein, M., Aja, G., van Wyk, B., Bosch-Capblanch, X., & Patrick, M. (2005). Lay health workers in primary and community health care. *The Cochrane Database of Systematic Reviews,**1*, CD004015. 10.1002/14651858.CD004015.pub2 10.1002/14651858.CD004015.pub2 15674924

[CR13] MacDougall, D., Halperin, B. A., Isenor, J., MacKinnon-Cameron, D., Li, L., McNeil, S. A., Langley, J. M., & Halperin, S. A. (2016). Routine immunization of adults by pharmacists: Attitudes and beliefs of the Canadian public and health care providers. *Human Vaccines & Immunotherapeutics,**12*(3), 623–631. 10.1080/21645515.2015.1093714 26810485 10.1080/21645515.2015.1093714 PMC4964643

[CR14] Office of the Auditor General of Ontario. (2022). *Value-for-money audit: COVID-19 Vaccination Program*. Retrieved January 4, 2024, from https://www.auditor.on.ca/en/content/annualreports/arreports/en22/AR_COVIDVaccination_en22.pdf

[CR15] Orkin, A. M., McArthur, A., Venugopal, J., Kithulegoda, N., Martiniuk, A., Buchman, D. Z., Kouyoumdjian, F., Rachlis, B., Strike, C., & Upshur, R. (2019). Defining and measuring health equity in research on task shifting in high-income countries: A systematic review. *SSM - Population Health,**7*, 100366. 10.1016/j.ssmph.2019.100366 30886887 10.1016/j.ssmph.2019.100366 PMC6402379

[CR16] Orkin, A. M., Rao, S., Venugopal, J., Kithulegoda, N., Wegier, P., Ritchie, S. D., VanderBurgh, D., Martiniuk, A., Salamanca-Buentello, F., & Upshur, R. (2021). Conceptual framework for task shifting and task sharing: An international Delphi study. *Human Resources for Health,**19*(1), 61. 10.1186/s12960-021-00605-z 33941191 10.1186/s12960-021-00605-z PMC8091141

[CR17] Professional Practice Behaviour for all Health Professional Students. (2015). University of Toronto Office of the Governing Council [March 31, 2015]. Retrieved January 4 2024, from https://governingcouncil.utoronto.ca/secretariat/policies/professional-practice-behaviour-all-health-professional-students-standards-0

[CR18] Regulated Health Professions Act. (1991). Ontario Ministry of Health Regulated Health Professions Act, *1991, S.O. 1991, c. 18*. Ontario.Ca. Retrieved January 2024, from https://www.ontario.ca/laws/view

[CR19] Sell, H., Assi, A., Driedger, S. M., Dubé, È., Gagneur, A., Meyer, S. B., Robinson, J., Sadarangani, M., Tunis, M., & MacDonald, S. E. (2021). Continuity of routine immunization programs in Canada during the COVID-19 pandemic. *Vaccine,**39*(39), 5532–5537. 10.1016/j.vaccine.2021.08.044 34426028 10.1016/j.vaccine.2021.08.044 PMC8439618

[CR20] Taddio, A., Gudzak, V., Jantzi, M., Logeman, C., Bucci, L. M., MacDonald, N. E., & Moineddin, R. (2022). Impact of the CARD (Comfort Ask Relax Distract) system on school-based vaccinations: A cluster randomized trial. *Vaccine,**40*(19), 2802–2809. 10.1016/j.vaccine.2022.02.069 35365344 10.1016/j.vaccine.2022.02.069

[CR21] Tetui, M., Tennant, R., Patten, A., Giilck, B., Burns, C. M., Waite, N., & Grindrod, K. (2023). Role satisfaction among community volunteers working in mass COVID-19 vaccination clinics, Waterloo Region. *Canada. BMC Public Health,**23*(1), 1199. 10.1186/s12889-023-15597-9 37344794 10.1186/s12889-023-15597-9 PMC10283305

[CR22] Unregulated Healthcare Professionals. (2023). NZ Immunisation Advisory Centre. Retrieved September 30, 2023, from https://www.immune.org.nz/immunisation-workforce/unregulated-healthcare-professionals

