# Supplementary material for: Effectiveness, acceptability, and potential of lay student vaccinators to improve vaccine delivery
Source: Can J Public Health. 2024 Jul 17;115(5):746–55. doi: 10.17269/s41997-024-00909-2 (PMC11534912; doi:10.17269/s41997-024-00909-2)
Supplement: Supplementary file 1 — Supplementary file1 (PDF 139 KB) [file 41997_2024_909_MOESM1_ESM.pdf]

Title:

Effectiveness, acceptability, and potential of lay student vaccinators to improve vaccine delivery

Authors:

Ryan Yee, MASc[1] *(ORCiD 0000-0003-3744-8530)*
Cécile Raymond, RN, MHSc[2]
Meredith Strong, BSc[3]
Lori Seeton, MHA[2]
Akash Kothari, MSc[1]
Victor Lo, MASc[1]
Emma-Cole McCubbin, MEd[1]
Alexandra Kubica, MPH[4]
Anna Subic, MPH[4]
Anna Taddio, PhD, MSc[5]
Mohammed Mall, BSc[6]
Sheikh Noor Ul Amin, MD[1]
Monique Martin, MD[1]
Aaron M. Orkin, MD, MSc, MPH, PhD[4,7-9+] *(ORCiD 0000-0002-1111-8720)*

1. University of Toronto Emergency First Responders, University of Toronto, Toronto, Canada
2. University Health Network, Toronto, Canada
3. Office of the Vice-Provost, Students, University of Toronto, Toronto, Canada
4. Dalla Lana School of Public Health, University of Toronto, Toronto, Canada
5. Leslie Dan Faculty of Pharmacy, University of Toronto, Toronto, Canada
6. West Toronto Ontario Health Team, Toronto, Canada
7. Department of Family and Community Medicine, University of Toronto, Toronto, Canada
8. Department of Emergency Medicine, St. Joseph's Health Centre, Unity Health Toronto, Toronto, Canada
9. Li Ka Shing Knowledge Institute of Unity Health Toronto, Toronto, Canada

\+ Corresponding author, aaron.orkin@utoronto.ca

Supplement 1: Medical Directives

Supplement 1: Medical Directives

Medical Directive

Title: Delegation of COVID-19 Immunization to University of Toronto Emergency First Responders

Activation Date: ____
Version: ____

Sponsoring/Contact Person(s)

Names, titles, and emails of physicians/authorizers

Schedule of Revisions

Version	Revision Summary
1.0	Original

Order and/or delegated procedure:

- Intramuscular administration of COVID-19 vaccine
- Documentation in COVAX system
- Post-immunization observation for adverse events

Rationale

This directive authorizes the delegation of intramuscular injections for the purpose of administering the COVID-19 vaccine to individuals who are not regulated health care professionals as understood under the *Regulated Health Professions Act (RHPA).* The delegation of this procedure is authorized for the purpose of engaging the University of Toronto Emergency First Responders as COVID-19 immunizers. This directive authorizes members of the University of Toronto Emergency First Responders (UTEFR Member) to administer the COVID-19 vaccine under specific circumstances.

Although this directive is prompted by the December 2021 regulatory changes to the RHPA, it is not predicated on these changes, and is supported and enabled by the CPSO Policy on Delegation of Controlled Acts and would remain in force even in the event that the above regulatory changes to the RHPA were no longer in effect.

Authorized Implementers:

A UTEFR Member will be authorized to administer the COVID-19 vaccine and document in COVAX if and only if all of the following conditions are met:

a) The UTEFR Member has completed training on intramuscular injection technique and COVAX utilization and has received their training certification that is valid and on-file with the UTEFR Executive Director. Certification is valid for 90 days from completion of training, and renews for 90 days after each time the member participates in a vaccination clinic.
b) The UTEFR Member has demonstrated the skill of correctly administering an intramuscular injection in the presence of an MD, NP, RN, RPN or PharmD.
c) An MD, NP, RN, RPN, or PharmD is present at the vaccine clinic and has authorized the UTEFR Volunteer to administer vaccine under their supervision.

d) An MD, NP, RN, RPN, or PharmD has prepared the appropriate vaccine for administration.
e) The UTEFR Volunteer is confident in their ability to safely administer the vaccine and document in COVAX.

Training and certification as a lay vaccinator authorizes that individual to administer COVID-19 vaccine under the direct supervision of an MD, NP, RN, RPN or PharmD. This training and certification does not confer any authority to administer COVID-19 vaccine in any other context, with any other organization, or perform any other controlled act.

A list of authorized implementers is provided as appendix A.

Eligible Patients
A UTEFR Volunteer may only provide vaccination to a patient who meets all of the following criteria:

a) The patient receiving the vaccine is 16 years or older.
b) The patient has provided consent to receive the vaccine.
c) The patient is eligible for COVID-19 vaccination according to applicable Ontario guidelines.

The implementor may NOT vaccinate any individual who is their own first-degree relative, household contact, or a person with whom they have another close relationship.

In any instance where there is a medical emergency or the patient receiving the vaccine has any complex needs requiring additional monitoring, the MD, NP, RN, RPN or PharmD will provide the vaccine and any post-vaccination monitoring and aftercare to the patient. If the UTEFR Member has any concerns about vaccinating a patient, they should immediately notify the health professional staff on site and refrain from vaccinating the patient.

Procedure:
COVAX Registration and Documentation:
a) Register the patient in COVAX
b) Complete pre-vaccination checklist as prompted by COVAX
c) Document immunization administration in COVAX

Immunization:
d) Expose the upper arm and deltoid of the client's preferred arm (usually the non-dominant arm).
e) Cleanse the skin with an alcohol-based prep pad or equivalent skin preparation product.
f) Administer the provided intramuscular injection in deltoid muscle of the client's upper arm.
g) Dispose of the needle and syringe in a provided sharps container.
h) Apply an adhesive bandage to the injection site as needed.
i) Observe the client after immunization and alert the supervising health professional to any adverse events.
j) Communicate to the supervising health professional that the immunization was delivered.

Review Schedule:
This directive shall be reviewed and renewed quarterly. If the directive has not been signed within 90 days prior it is null and void.

Authorizer Approval

Name of Physician or Authorizer	Signature	Date

Appendix A
Implementer Approval Form

Name of Implementer	Signature	Date

Medical Directive

Title:
Delegation of Influenza Immunization to
University of Toronto Emergency First Responders

Activation Date: ____
Version: ____

Sponsoring/Contact Person(s)

Names, titles, and emails of physicians/authorizers

Schedule of Revisions

Version	Revision Summary
1.0	Original

Order and/or delegated procedure:

- Intramuscular administration of Influenza vaccine
- Post-immunization observation for adverse events

Rationale

This directive authorizes the delegation of intramuscular injections for the purpose of administering the influenza vaccine to individuals who are not regulated health care professionals as understood under the *Regulated Health Professions Act (RHPA).* The delegation of this procedure is authorized for the purpose of engaging the University of Toronto Emergency First Responders as immunizers. This directive authorizes members of the University of Toronto Emergency First Responders (UTEFR Member) to administer the influenza vaccine under specific circumstances.

Authorized Implementers:

A UTEFR Member will be authorized to administer the influenza vaccine and document on the patient's vaccination record if and only if all of the following conditions are met:

a) The UTEFR Member has completed training on intramuscular injection technique and documentation and has received their training certification that is valid and on-file with the UTEFR Executive Director. Certification is valid for 90 days from completion of training, and renews for 90 days after each time a UTEFR Member participates in a vaccination clinic.
b) The UTEFR Member has demonstrated the skill of correctly administering an intramuscular injection in the presence of an MD, NP, RN, RPN or PharmD.
c) An MD, NP, RN, RPN, or PharmD is present at the vaccine clinic and has authorized the UTEFR Member to administer vaccine under their supervision.
d) An MD, NP, RN, RPN, or PharmD has prepared the appropriate vaccine for administration.
e) The UTEFR Member is confident in their ability to safely administer the vaccine and document accordingly

Training and certification as a lay vaccinator authorizes that individual to administer the influenza vaccine under the direct supervision of an MD, NP, RN, RPN or PharmD. This training and certification does not confer any authority to administer influenza vaccine or any other vaccine in any other context, with any other organization, or perform any other controlled act.

A list of authorized implementers is provided as appendix A.

Eligible Patients
A UTEFR Member may only provide vaccination to a patient who meets all of the following criteria:

a) The patient receiving the vaccine is 16 years or older.
b) The patient has provided consent to receive the vaccine.
c) The patient is eligible for influenza vaccination according to applicable Ontario guidelines.

The implementor may NOT vaccinate any individual who is their own first-degree relative, household contact, or a person with whom they have another close relationship.

In any instance where there is a medical emergency or the patient receiving the vaccine has any complex needs requiring additional monitoring, the MD, NP, RN, RPN or PharmD will provide the vaccine and any post-vaccination monitoring and aftercare to the patient. If the UTEFR Member has any concerns about vaccinating a patient, they should immediately notify the health professional staff on site and refrain from vaccinating the patient.

Procedure:
Immunization:

a) Expose the upper arm and deltoid of the client's preferred arm (usually the non-dominant arm).
b) Cleanse the skin with an alcohol-based prep pad or equivalent skin preparation product.
c) Administer the provided intramuscular injection in deltoid muscle of the client's upper arm.
d) Dispose of the needle and syringe in a provided sharps container.
e) Apply an adhesive bandage to the injection site as needed.
f) Observe the client after immunization and alert the supervising health professional to any adverse events.
g) Communicate to the supervising health professional that the immunization was delivered.

Documentation:

h) Document the vaccination on the patient's vaccination slip or card.
i) Deliver the vaccination card or slip to the patient.

Review Schedule:
This directive shall be reviewed and renewed quarterly. If the directive has not been signed within 90 days prior it is null and void.

Authorizer Approval

Name of Physician or Authorizer	Signature	Date

Appendix A
Implementer Approval Form

Name of Implementer	Signature	Date
