# Supplementary material for: Effectiveness, acceptability, and potential of lay student vaccinators to improve vaccine delivery
Source: Can J Public Health. 2024 Jul 17;115(5):746–55. doi: 10.17269/s41997-024-00909-2 (PMC11534912; doi:10.17269/s41997-024-00909-2)
Supplement: Supplementary file 2 — Supplementary file2 (PDF 160 KB) [file 41997_2024_909_MOESM2_ESM.pdf]

Title:
Effectiveness, acceptability, and potential of lay student vaccinators to improve vaccine delivery

Authors:
Ryan Yee, MASc[1] *(ORCiD 0000-0003-3744-8530)*
Cécile Raymond, RN, MHSc[2]
Meredith Strong, BSc[3]
Lori Seeton, MHA[2]
Akash Kothari, MSc[1]
Victor Lo, MASc[1]
Emma-Cole McCubbin, MEd[1]
Alexandra Kubica, MPH[4]
Anna Subic, MPH[4]
Anna Taddio, PhD, MSc[5]
Mohammed Mall, BSc[6]
Sheikh Noor Ul Amin, MD[1]
Monique Martin, MD[1]
Aaron M. Orkin, MD, MSc, MPH, PhD[4,7-9+] *(ORCiD 0000-0002-1111-8720)*

1. University of Toronto Emergency First Responders, University of Toronto, Toronto, Canada
2. University Health Network, Toronto, Canada
3. Office of the Vice-Provost, Students, University of Toronto, Toronto, Canada
4. Dalla Lana School of Public Health, University of Toronto, Toronto, Canada
5. Leslie Dan Faculty of Pharmacy, University of Toronto, Toronto, Canada
6. West Toronto Ontario Health Team, Toronto, Canada
7. Department of Family and Community Medicine, University of Toronto, Toronto, Canada
8. Department of Emergency Medicine, St. Joseph's Health Centre, Unity Health Toronto, Toronto, Canada
9. Li Ka Shing Knowledge Institute of Unity Health Toronto, Toronto, Canada

\+ Corresponding author, aaron.orkin@utoronto.ca

Supplement 2: Survey Questions

UTEFR Lay Vaccination Clinic

Start of Block: Letter of Information and Consent

Informed Consent Letter of Information/Consent

Background:
The Dalla Lana School of Public Health and the University of Toronto Emergency First Responders (UTEFR) have launched a collaboration to develop and evaluate a series of COVID-19 vaccination clinics operated by lay providers (i.e. non-regulated health professionals).

Are there any risks to doing this study?
Participation in this study will involve completion of this digital survey, posing minimal to no risk. You can decline to answer any questions. You can also choose to withdraw from this study at any time. Completion of this survey is voluntary and will not affect the care received or your role at the clinic.

Are there any benefits to participating in this survey?
There are no immediate benefits to taking part in this study. However, this project aims to explore perspectives on the use of lay vaccinators and has the potential to influence policy makers and future models of public health. The overarching goal of this project is to demonstrate that lay providers can safely and efficiently operate a vaccine clinic, thereby allowing more physicians, nurses or pharmacists to perform more complex assessments that are restricted to their professions. Therefore, participation in this study may be beneficial in terms of creating a new vaccination model in Canada that optimally re-distributes health and human resources.

How will my responses be shared with the public?
Every effort will be made to protect your confidentiality and only non-personally identifiable information will be included in any research publication. Questions have been designed to allow you to respond anonymously, with the exception of if you would like to be contacted by a research staff member. Data will be stored and retained for future analyses on our University SharePoint system, which is encrypted and password protected.

Questions about the study?
If you have any questions or need more information about the study itself, please contact our Principal Investigator Dr. Aaron Orkin aaron.orkin@mail.utoronto.ca. This study has been reviewed by the University of Toronto Research Ethics Board and received ethics clearance. If you have concerns or questions about your rights as a participant or about the way the study is conducted, please contact: Research Oversight and Compliance Office – Human Research

Ethics Program ethics.review@utoronto.ca 416-946-3273

Thank you.

◯ I understand and consent to participating in this research survey (1)

End of Block: Letter of Information and Consent

Start of Block: Demographic

Please indicate your role at the clinic.

◯ Attendee (I got vaccinated) (1)

◯ Vaccinator (UTEFR Member) (2)

◯ Staff or Supervisor (UHN or UofT Staff) (3)

Did you have an important experience at the lay vaccinator clinic and would like to be contacted to discuss it with an evaluator?

◯ Yes, I would like to be contacted by an evaluator (1)

◯ No, I do not need to be contacted (2)

Display This Question:

If Did you have an important experience at the lay vaccinator clinic and would like to be contacted... = Yes, I would like to be contacted by an evaluator

What is your name? (Only necessary if you would like a follow up)

____

Display This Question:

If Did you have an important experience at the lay vaccinator clinic and would like to be contacted... = Yes, I would like to be contacted by an evaluator

Q5 What is your email address? (Only necessary if you would like a follow up)

____

Display This Question:

If Did you have an important experience at the lay vaccinator clinic and would like to be contacted... = Yes, I would like to be contacted by an evaluator

Q6 What is your phone number? (Only necessary if you would like a follow up)

____

Display This Question:

If Did you have an important experience at the lay vaccinator clinic and would like to be contacted... = Yes, I would like to be contacted by an evaluator

Q26 Please feel free to describe your experience here before we contact you for a follow up.

____

____

____

____

____

Page Break

Q8 Which age group do you fall into?

- ○ 0-17 (1)
- ○ 18-30 (2)
- ○ 31-60 (3)
- ○ 61-84 (4)
- ○ 85+ (5)
- ○ Prefer not to say (6)

Q27 What is your gender?

- ○ Male (1)
- ○ Female (2)
- ○ Other (3) ____
- ○ Prefer not to say (4)

End of Block: Demographic

Start of Block: This section primarily applies to those who were vaccinated by a lay provider.

Please indicate your agreement level with the following statements:

	Strongly disagree (1)	Somewhat disagree (2)	Neither agree nor disagree (3)	Somewhat agree (4)	Strongly agree (5)
I felt safe being vaccinated by a lay provider. (1)	○	○	○	○	○
I am comfortable with lay providers administering vaccines as long as they have appropriate training and supervision. (2)	○	○	○	○	○
Getting the vaccine from a lay provider felt the same as getting a vaccine from any other provider. (3)	○	○	○	○	○
The lay vaccinator made me feel comfortable. (4)	○	○	○	○	○
This vaccine clinic felt similar to other vaccine clinics that I have attended. (5)	○	○	○	○	○

I would feel comfortable with lay providers giving other vaccines, such as flu shots. (6)	○	○	○	○	○

Page Break

Do you have any concerns regarding the use of lay vaccinators?

◯ Yes (1)

◯ No (2)

Display This Question:

If Do you have any concerns regarding the use of lay vaccinators? = Yes

Please describe your concerns regarding the use of lay vaccinators.

____

____

____

____

____

Would you attend our clinic again, or another clinic run by lay providers?

◯ Yes (1)

◯ No (2)

◯ Other (3) ____

Would you recommend our clinic to your friends or family?

◯ Yes (1)

◯ No (2)

◯ Other (3) ____

Are there any changes you would like to see made for future vaccine clinics?

____

____

____

____

____

Please feel free to provide any additional comments or feedback here.

____

____

____

____

____

End of Block: This section primarily applies to those who were vaccinated by a lay provider.

Start of Block: This section only applies to UTEFR volunteers/vaccinators/lay providers.

Display This Question:

If Please indicate your role at the clinic. = Vaccinator (UTEFR Member)

Please rate your agreement level on the following statements with respect to your experience providing vaccinations:

	Strongly disagree (1)	Somewhat disagree (2)	Neither agree nor disagree (3)	Somewhat agree (4)	Strongly agree (5)
I felt comfortable administering the vaccine. (1)	○	○	○	○	○
The amount of training we received was enough to instil confidence in providing the vaccine. (2)	○	○	○	○	○
The supervising staff were approachable and able to answer my questions. (3)	○	○	○	○	○
I would feel comfortable getting vaccinated by another lay provider. (4)	○	○	○	○	○

Display This Question:

If Please indicate your role at the clinic. = Vaccinator (UTEFR Member)

Do you have any previous experience as a vaccinator?

◯ I have no previous vaccinator experience (1)

◯ I have previous vaccinator experience or am licensed/certified to provide IM injections (2)

◯ Other (3) ____

Display This Question:

If Please indicate your role at the clinic. = Vaccinator (UTEFR Member)

Is there anything else you would like us to know regarding your experience or perspective as a lay vaccinator?

____

____

____

____

____

End of Block: This section only applies to UTEFR volunteers/vaccinators/lay providers.

Start of Block: This section only applies to UHN or UofT staff.

Display This Question:

If Please indicate your role at the clinic. = Staff or Supervisor (UHN or UofT Staff)

What was your staff role at our clinic?

◯ Registration staff (1)

◯ Supervising manager or regulated health provider (2)

Display This Question:

If What was your staff role at our clinic? = Supervising manager or regulated health provider

What is your profession or health care background?

○ Nurse (RN, RPN or NP) (1)

○ Pharmacist (PharmD) (2)

○ Physician (MD) (3)

○ Other (4) ____

Display This Question:

If Please indicate your role at the clinic. = Staff or Supervisor (UHN or UofT Staff)

Please rate your agreement level on the following statements with respect to your experience supporting the vaccine clinic:

	Strongly Disagree (1)	Somewhat disagree (2)	Neither agree nor disagree (3)	Somewhat agree (4)	Strongly agree (5)
I felt comfortable working with and supervising lay vaccinators. (1)	○	○	○	○	○
The training provided to lay vaccinators was sufficient. (2)	○	○	○	○	○
I would feel comfortable supervising more lay vaccinators at a future clinic. (3)	○	○	○	○	○
Lay providers should be allowed to work at any vaccine clinic such that they have the appropriate training. (4)	○	○	○	○	○

Display This Question:

If What was your staff role at our clinic? = Supervising manager or regulated health provider

What is the maximum number of lay providers you would feel comfortable supervising? (i.e. 1 supervisor to ___ lay vaccinators)

____

Display This Question:

If Please indicate your role at the clinic. = Staff or Supervisor (UHN or UofT Staff)

Is there anything else you would like us to know regarding your experience or perspective on the supervision of lay vaccinators?

____

____

____

____

____

End of Block: This section only applies to UHN or UofT staff.
