# Supplementary material for: Effectiveness, acceptability, and potential of lay student vaccinators to improve vaccine delivery
Source: Can J Public Health. 2024 Jul 17;115(5):746–55. doi: 10.17269/s41997-024-00909-2 (PMC11534912; doi:10.17269/s41997-024-00909-2)
Supplement: Supplementary file 3 — Supplementary file3 (PDF 206 KB) [file 41997_2024_909_MOESM3_ESM.pdf]

**Title:**
Effectiveness, acceptability, and potential of lay student vaccinators to improve vaccine delivery

**Authors:**
*Ryan Yee, MASc[1] (ORCiD 0000-0003-3744-8530)*
*Cécile Raymond, RN, MHSc[2]*
*Meredith Strong, BSc[3]*
*Lori Seeton, MHA[2]*
*Akash Kothari, MSc[1]*
*Victor Lo, MASc[1]*
*Emma-Cole McCubbin, MEd[1]*
*Alexandra Kubica, MPH[4]*
*Anna Subic, MPH[4]*
*Anna Taddio, PhD, MSc[5]*
*Mohammed Mall, BSc[6]*
*Sheikh Noor Ul Amin, MD[1]*
*Monique Martin, MD[1]*
*Aaron M. Orkin, MD, MSc, MPH, PhD[4,7-9+] (ORCiD 0000-0002-1111-8720)*

1. University of Toronto Emergency First Responders, University of Toronto, Toronto, Canada
2. University Health Network, Toronto, Canada
3. Office of the Vice-Provost, Students, University of Toronto, Toronto, Canada
4. Dalla Lana School of Public Health, University of Toronto, Toronto, Canada
5. Leslie Dan Faculty of Pharmacy, University of Toronto, Toronto, Canada
6. West Toronto Ontario Health Team, Toronto, Canada
7. Department of Family and Community Medicine, University of Toronto, Toronto, Canada
8. Department of Emergency Medicine, St. Joseph's Health Centre, Unity Health Toronto, Toronto, Canada
9. Li Ka Shing Knowledge Institute of Unity Health Toronto, Toronto, Canada

\+ Corresponding author, aaron.orkin@utoronto.ca

## Supplement 3: Lay Vaccinator Survey CHERRIES Table

**Supplementary Table 1.** Lay vaccinator demonstration project survey checklist for reporting internet e-surveys (CHERRIES).

| *Item Category* | *Checklist Item* | *Explanation* |
|---|---|---|
| **Design** | Describe survey design | All clinic patients and providers were invited to complete the voluntary survey using a QR code. Patients and providers involved in UTEFR lay vaccinator clinics were invited to complete a voluntary Qualtrics survey (Qualtrics Provo UT) using a QR code displayed in the post-vaccination monitoring area. |
| **IRB (Institutional Review Board) approval and informed consent process** | IRB approval | All procedures performed in studies were in accordance with the standards of the University of Toronto Research Ethics Board (Human Ethics Protocol #43500). |
| | Informed consent | Information on informed consent was explained on page 1 of the survey and documented electronically with a checkbox. Participants were allowed to withdraw from the study at any time. |
| | Data protection | Data without identifiable personal information was stored and protected on the University's encrypted OneDrive. Only involved researchers were able to access the data. |
| **Development and pre-testing** | Development and testing | Investigators reviewed and revised the survey questions until consensus. Three basic question types were used to simplify the participant experience: 1) Likert scales (5-point agreement, positively worded statements); 2) yes/no/other questions; 3) open-text questions. Investigators tested the survey with demo QR codes and received IRB approval prior to survey release. |
| **Recruitment process and description of the sample having** | Open survey versus closed survey | An open survey design permitted all clinic visitors to participate using their own mobile device or a tablet provided by the clinic. |

| | | |
|---|---|---|
| **access to the questionnaire** | | |
| | Contact mode | The lay vaccinator program was advertised to vaccinator candidates in a UTEFR WhatsApp group chat comprised of 80+ members between March 2022 – February 2023. 30 students requested COVID Critical Care eLearning accounts to begin online asynchronous training. 27 students completed all online requirements and proceeded to in-person training and became vaccinators. Regulated providers were scheduled to clinics by the UHN as per usual practices. Investigators made initial contact with vaccinators and regulated providers and invited them to complete the 'vaccinator' and 'supervisor' survey sections respectively. Clinic patients made initial contact with investigators or clinic providers and were invited to complete the patient feedback survey after their visit to the clinic. |
| | Advertising the survey | Surveys were advertised at clinics with a QR code displayed in the post-vaccination waiting area. Clinics were advertised independent of surveys on the University's UTogether vaccine campaign website, through email to students living in residence, and on the UTEFR Instagram page. Off-campus flu vaccine clinics were advertised by the community health centres as per annual campaigns. |
| **Survey administration** | Web/E-mail | The Qualtrics survey platform automatically collected responses over the internet. |
| | Context | This survey was offered to all clinic attendees, who may or may not hold different or more positive views towards vaccination than the general population. The survey did not include questions about what vaccine the patient received or their baseline fears towards vaccination. |
| | Mandatory/voluntary | Completing the survey was voluntary and did not affect a participant's role or care received at the clinic. |

| | | |
|---|---|---|
| | Incentives | No direct incentives were offered for completing the survey. Benefits from attending the clinic were independent of the survey included obtaining immunization status or vaccine documentation, complying with public health or university regulations, or obtaining a free COVID-19 rapid-antigen testing kit. |
| | Time/Date | Data collection began with our third clinic in October 2022 and completed with our eleventh clinic in February 2023. |
| | Randomization of items or questionnaires | Questions were not randomized or alternated across surveys. |
| | Adaptive questioning | Adaptive questioning was used to simplify questions and reduce complexity (e.g., an open-text box asking participants to describe concerns would appear if participants first checked off yes to having concerns). |
| | Number of Items | One to six items per page. |
| | Number of screens (pages) | Six pages for clinic attendees/patients, seven for vaccinators, eight for staff/supervisors.. |
| | Completeness check | Incomplete questionnaires were excluded from data analysis. There were no non-response options, but respondents were able to skip any question. |
| | Review step | Respondents were able to review and change their answers through a Back button. |
| **Response rates** | Unique site visitor | 199 unique site visitors (total # of clinic attendees based on 162 eligible patients, 27 lay vaccinators, and 10 supervisors). |

| | | |
|---|---|---|
| | View rate (Ratio of unique first survey page visitors/unique site visitors) | 199/199 = 100% (QR was shown to all unique site visitors in post-vaccine area). |
| | Participation rate (Ratio of unique visitors who agreed to participate/unique first survey page visitors) | 157 / 199 = 78.9% |
| | Completion rate (Ratio of users who finished the survey/users who agreed to participate) | 141 / 157 = 89.8% |
| **Preventing multiple entries from the same individual** | Cookies used | The survey did not collect cookies. |
| | IP check | IP addresses were identified, and duplicates removed before data analysis by keeping the most complete entry. |
| | Log file analysis | There was no other log file analysis for this survey. |
| | Registration | This survey did not require registration for completion. |
| **Analysis** | Handling of incomplete questionnaires | Only completed questionnaires were analyzed. |
| | Questionnaires submitted with an atypical timestamp | There was no identification of questionnaires with atypical timestamps (e.g., submitted ≥ 24hrs after clinics). The QR code was available to vaccinators after clinics if requested.. |
| | Statistical correction | No statistical correction methods were used. |
