# Supplementary material for: Effectiveness, acceptability, and potential of lay student vaccinators to improve vaccine delivery
Source: Can J Public Health. 2024 Jul 17;115(5):746–55. doi: 10.17269/s41997-024-00909-2 (PMC11534912; doi:10.17269/s41997-024-00909-2)
Supplement: Supplementary file 4 — Supplementary file4 (PDF 129 KB) [file 41997_2024_909_MOESM4_ESM.pdf]

Title:
Effectiveness, acceptability, and potential of lay student vaccinators to improve vaccine delivery

Authors:
Ryan Yee, MASc[1] (ORCiD 0000-0003-3744-8530)
Cécile Raymond, RN, MHSc[2]
Meredith Strong, BSc[3]
Lori Seeton, MHA[2]
Akash Kothari, MSc[1]
Victor Lo, MASc[1]
Emma-Cole McCubbin, MEd[1]
Alexandra Kubica, MPH[4]
Anna Subic, MPH[4]
Anna Taddio, PhD, MSc[5]
Mohammed Mall, BSc[6]
Sheikh Noor Ul Amin, MD[1]
Monique Martin, MD[1]
Aaron M. Orkin, MD, MSc, MPH, PhD[4,7-9+] (ORCiD 0000-0002-1111-8720)

1. University of Toronto Emergency First Responders, University of Toronto, Toronto, Canada
2. University Health Network, Toronto, Canada
3. Office of the Vice-Provost, Students, University of Toronto, Toronto, Canada
4. Dalla Lana School of Public Health, University of Toronto, Toronto, Canada
5. Leslie Dan Faculty of Pharmacy, University of Toronto, Toronto, Canada
6. West Toronto Ontario Health Team, Toronto, Canada
7. Department of Family and Community Medicine, University of Toronto, Toronto, Canada
8. Department of Emergency Medicine, St. Joseph's Health Centre, Unity Health Toronto, Toronto, Canada
9. Li Ka Shing Knowledge Institute of Unity Health Toronto, Toronto, Canada

\+ Corresponding author, aaron.orkin@utoronto.ca

Supplement 4: Survey Demographics

Supplementary Table 2. Lay vaccinator clinic survey demographics and participation (n=141).

	Patients	Lay vaccinators	Supervisors
Participation			
Surveys offered	162	27	10
Surveys completed	122	17	2
Gender			
Female	79	6	1
Male	42	11	1
Other	1	0	0
Age			
≤17	6	1	0
18–30	104	16	1
31–60	12	0	1
>60	0	0	0

Note: Response rates were calculated by dividing surveys completed by surveys offered for each participant type. Of the 293 doses administered, 40 participants received both an influenza and COVID-19 vaccine at the same time. 91 doses were given at 4 clinics where the survey was not administered.
